# A Novel Serum-Based Bioassay for Quantification of Cancer-Associated Transformation Activity: A Case–Control and Animal Study

**DOI:** 10.3390/diagnostics15151975

**Published:** 2025-08-06

**Authors:** Aye Aye Khine, Hsuan-Shun Huang, Pao-Chu Chen, Chun-Shuo Hsu, Ying-Hsi Chen, Sung-Chao Chu, Tang-Yuan Chu

**Affiliations:** 1 Center for Prevention and Therapy of Gynecological Cancers, Department of Research, Hualien Tzu Chi Hospital, Hualien 97004, Taiwan; aye2224@gmail.com (A.A.K.); huang.sansam@gmail.com (H.-S.H.); 2 Department of Obstetrics & Gynecology, Hualien Tzu Chi Hospital, Hualien 97004, Taiwan; 3 Department of Obstetrics & Gynecology, Dalin Tzu Chi Hospital, Dalin 90749, Taiwan; 4 Institute of Medical Science, Tzu Chi University, Hualien 97004, Taiwan; 5 Department of Hematology and Oncology, Hualien Tzu Chi Hospital, Buddhist Tzu Chi Medical Foundation, Hualien 97004, Taiwan; 6 School of Medicine, College of Medicine, Tzu Chi University, Hualien 97004, Taiwan

**Keywords:** cancer diagnosis, anchorage-independent growth, blood test, biological assay, ovarian neoplasms

## Abstract

**Background/Objectives**: The detection of ovarian cancer remains challenging due to the lack of reliable serum biomarkers that reflect malignant transformation rather than mere tumor presence. We developed a novel biotest using an immortalized human fallopian tube epithelial cell line (TY), which exhibits anchorage-independent growth (AIG) in response to cancer-associated serum factors. **Methods**: Sera from ovarian and breast cancer patients, non-cancer controls, and ID8 ovarian cancer-bearing mice were tested for AIG-promoting activity in TY cells. **Results**: TY cells (passage 96) effectively distinguished cancer sera from controls (68.50 ± 2.12 vs. 17.50 ± 3.54 colonies, *p* < 0.01) and correlated with serum CA125 levels (*r* = 0.73, *p* = 0.03) in ovarian cancer patients. Receiver operating characteristic (ROC) analysis showed high diagnostic accuracy (AUC = 0.85, cutoff: 23.75 colonies). The AIG-promoting activity was mediated by HGF/c-MET and IGF/IGF-1R signaling, as inhibition of these pathways reduced phosphorylation and AIG. In an ID8 mouse ovarian cancer model, TY-AIG colonies strongly correlated with tumor burden (*r* = 0.95, *p* < 0.01). **Conclusions**: Our findings demonstrate that the TY cell-based AIG assay is a sensitive and specific biotest for detecting ovarian cancer and potentially other malignancies, leveraging the fundamental hallmark of malignant transformation.

## 1. Introduction

Despite advances in cancer screening, approximately 50% of malignancies are still detected at advanced stages. While established screening programs have reduced mortality for cervical, breast, and colorectal cancers [1,2], most cancers—particularly those that are deeply located and rapidly disseminating—remain challenging to diagnose early. Ovarian cancer exemplifies this problem, as it is often asymptomatic until late stages and lacks reliable detection methods [3].

Blood-based testing represents the most non-invasive and widely acceptable approach for cancer diagnosis. However, only a few malignancies—such as multiple myeloma [4], gestational trophoblastic diseases [5], and germ cell tumors [6]—have tumor markers with sufficient diagnostic utility. For ovarian cancer, current biomarkers like carbohydrate antigen 125 (CA125) and human epididymis protein 4 (HE4) suffer from low specificity and sensitivity, leading to high false-positive rates [7]. Notably, large-scale screening trials using these markers failed to reduce ovarian cancer mortality [8,9], and HE4 provided no additional diagnostic benefit over CA125 [10,11].

A fundamental limitation of existing tests is their reliance on surrogate markers rather than assessing the biological hallmarks of cancer. An ideal diagnostic assay should directly measure malignant potential, such as the capacity for anchorage-independent growth (AIG), a defining feature of transformed cells. Unlike normal cells, which undergo anoikis when detached, cancer cells acquire the ability to survive and proliferate without attaching to a solid surface. A biotest that can measure the AIG-promoting activity in serum may offer a functional readout of malignancy and an idea blood test for cancer diagnosis.

We previously established an immortalized human cell line, TY, from the fallopian tube fimbria epithelium, named for its developer, Professor Tang-Yuan Chu. TY cells form AIG colonies in soft agar in response to various transforming signals, including ovulatory follicular fluid [12]. Key mediators of this process—such as insulin-like growth factor 2 (IGF-2), hepatocyte growth factor (HGF), and epidermal growth factor receptor ligands—drive malignant transformation [13,14,15,16]. Intriguingly, TY cells also responded to sera from cancer patients, suggesting their potential utility for tumor-derived factors.

In this retrospective study, we aimed to validate the diagnostic performance of the TY-based AIG assay for ovarian cancer detection, optimize testing conditions to maximize sensitivity and specificity, and determine whether AIG activity correlates with tumor burden in preclinical models. By focusing on the functional hallmark of malignancy—rather than indirect markers—this biotest holds promise for improving early cancer detection.

## 2. Materials and Methods

### 2.1. Serum Samples

Sera ovarian cancer patients (*n* = 8) and normal controls (8 female and 8 male) were provided by the Tzuchi Joint Tissue Bank of Gynecological Oncology (supported by Buddhist Tzuchi Medical Foundation, TCMMP104, and Hualien Tzuchi General hospital, TCRD113-075). Sera of breast cancer patients (*n* = 8) were provided by the National Biobank Consortium of Taiwan (NBCT, No. 230115). Follicular fluids were collected from women undertaking oocyte retrieval for in vitro fertilization program in Hualien Tzuchi Hospital. The Research Ethics Committee of Hualien Tzuchi Hospital (IRB101-09, IRB112-241-B) approved the study. Informed consent was received from all individuals and patients.

Serum samples were prepared before scheduled surgery or during OPD visit. FFs devoid of blood or flush medium contamination were collected at the time of sono-guided transvaginal oocyte retrieval as described earlier [12]. Sera from 8–10 week old 5 female C57BL/6 mice were collected soon after post-euthanasia. All the serum and FF samples were aliquoted into 1.5 mL Eppendorf and stored at −80 °C, and were used for this study from 2022 January to 2024 June.

### 2.2. Cell Culture

Immortalized fallopian tube epithelium cells FT282-CCNE1 (a kind gift from Dr. Ronny Drapkin) [17], were maintained in MCDB105 and M199 media (Sigma, St. Louis, MO, USA) supplemented with 10% fetal bovine serum (FBS) and 100 IU/mL of penicillin, and 100 μg/mL of streptomycin. The human high-grade serous carcinoma cell line OVSAHO was cultured in RPMI-1640 medium with 10% FBS, 100 IU/mL of penicillin, and 100 μg/mL of streptomycin. MDA-MB-231 human breast adenocarcinoma cells were cultured in Dulbecco’s modified Eagle’s medium (DMEM, Sigma, USA) supplemented with 10% FBS, 100 IU/mL of penicillin, and 100 µg/mL of streptomycin. HCT116 human colon adenocarcinoma cells and A594 human non-small cell lung carcinoma cells were cultured in RPMI-1640 (Sigma, USA) medium with L-glutamine and sodium bicarbonate, supplemented with 10% FBS, 100 IU/mL of penicillin, and 100 µg/mL of streptomycin.

### 2.3. Development of TY Cells

To establish an immortalized human fallopian tube epithelial cell line, the fimbria tissue at distal fallopian tube was treated with 1% trypsin and 5 mM EDTA for 30 min to separate the epithelium. The peeled epithelium was then digested with collagenase (c2674, Sigma, USA) at 1.5 mg/mL for 1 h, and cultured in DMEM, 10% FBS, with 5 μg/mL insulin on a 0.1% gelatin-coated plate. At passage two, the primarily cultured fimbria epithelial cells were transduced with an HPV16 E6/E7 lentivirus and subsequently with a lentiviral *hTERT* (Applied Biological Materials Inc., Richmond, BC, Canada) at passage 20 to generate the TY cell line. The cells were maintained in the same way as the FT282-CCNE1 fallopian tube epithelium cells.

### 2.4. Anchorage Independent Growth (AIG) Assay

The AIG assay was performed using a two-layer soft agar system. For the base layer, 0.8% agarose was prepared by dissolving agarose powder in distilled water at 100 °C for 10 min, then mixed with an equal volume of serum-free medium to achieve a final 0.4% concentration. The upper layer containing cells was prepared by suspending 500 TY cells in 0.4% agarose (maintained at 37 °C) with serum-free medium. In each well of a 96-well plate, 50 µL of the 0.8% base agar was allowed to solidify before adding 50 µL of the cell-containing 0.4% upper agar mixed with test sera or ovulatory follicular fluid (FF) at a final concentration of 10%. Fresh serum/FF was replenished at 48 h, and serum-free medium was added every 72 h to maintain moisture. After 10 days of culture at 37 °C with 5% CO_2_, colonies >50 µm in diameter were quantified manually under phase-contrast microscopy. For inhibitor studies, TY cells were pretreated with 100 nM picropodophyllin (PPP) or 10 µM AMG-337 for 30 min prior to agar embedding.

### 2.5. Western Blot

Western blot analysis was performed by quantifying protein concentrations using Bradford reagent (Bio-Rad, #500-0006, Hercules, CA, USA), denaturing 30 μg of protein per sample in 4× Laemmli buffer at 95 °C for 5 min, followed by separation via SDS-PAGE and transfer to nitrocellulose membranes. Membranes were probed overnight at 4 °C with primary antibodies against c-MET (BS-0668R, Bioss, Wuhan, China), phospho-c-MET (#3077S), IGF-1Rβ (#3027S), phospho-IGF-1Rβ (#3918S) (all 1:1000 from Cell Signaling, Danvers, MA, USA), and β-actin (#4967S, 1:5000, Cell Signaling, USA) as loading control, followed by TBST washes and incubation with HRP-conjugated secondary antibodies. Protein bands were visualized using ECL detection reagent (GE Healthcare, RPN2209, Buckinghamshire, UK) and imaged.

### 2.6. ID8 Syngeneic Mouse Ovarian Cancer Model

The mouse ovarian cancer cell line ID8 (SCC145, Sigma-Aldrich, St. Louis, MO, USA) was maintained in high-glucose Dulbecco’s Modified Eagle Medium (DMEM) supplemented with 4% fetal bovine serum (FBS), 5 μg/mL insulin, 5 μg/mL transferrin, 5 ng/mL sodium selenite, 100 IU/mL penicillin, and 100 μg/mL streptomycin at 37 °C in a 5% CO_2_ atmosphere.

For tumor induction, 1 × 10^5^ ID8 cells suspended in 20 μL of serum-free medium were injected intraperitoneally into 8–10 week old female C57BL/6 mice. Four months post-injection, animals were euthanized. At necropsy, the peritoneal cavity was systematically examined and all visible tumor nodules were carefully dissected. Total tumor burden was quantified by measuring the combined weight of all excised tumors. Cardiac puncture was performed immediately post-euthanasia to collect blood samples, which were centrifuged at 4000× *g* for 30 min at 4 °C to obtain serum. Serum aliquots were stored at −80 °C until analysis. All animal procedures were approved by the Institutional Animal Care and Use Committee of Tzu Chi University (Protocol 112-30).

### 2.7. Statistical Analysis

GraphPad Prism (ver.8.0c) (GraphPad Software, San Diego, CA, USA) was applied to perform unpaired Student’s *t*-tests which were used to analysis the AIG count, and linear correlations were done by the Pearson correlation coefficient. *p* values less than 0.05 were defined as significant.

## 3. Results

### 3.1. Characteristics of TY Cells

**Figure 1:**
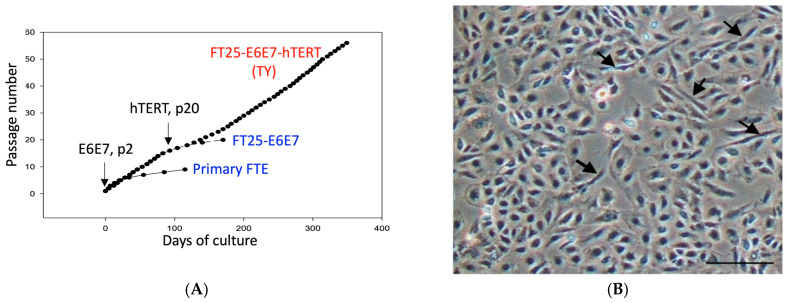

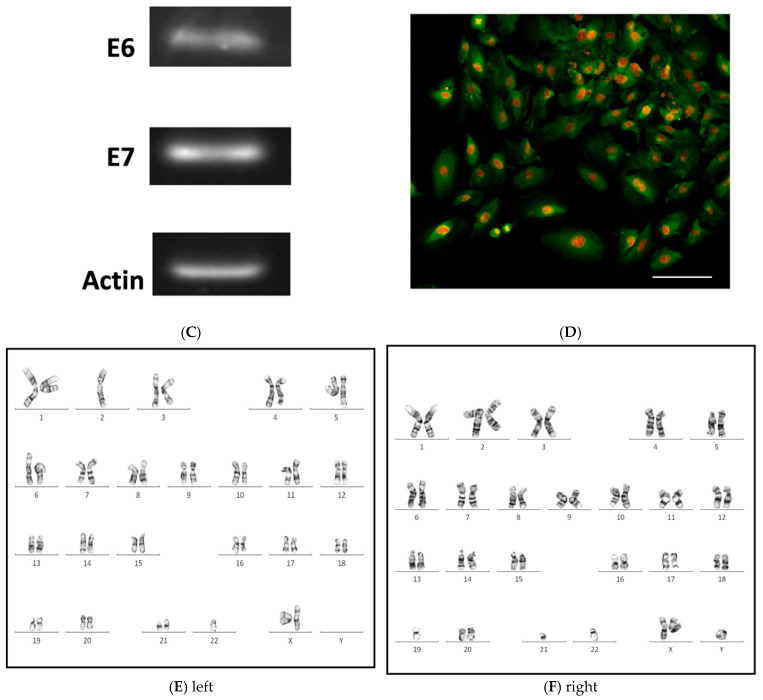
Generation and characterization of the immortalized TY cell line. (**A**) Schematic of TY cell development. Primary fallopian tube fimbrial epithelial cells (Primary FTE) were transduced with HPV16 E6/E7 at passage 2 (FT25-E6E7), followed by *hTERT* introduction at passage 20 (FT25-E6E7-hTERT). While primary and E6/E7-transduced cells underwent senescence, double-transduced cells (renamed TY) achieved immortal growth. (**B**,**D**) Phase-contrast (**B**) and immunofluorescence (**D**) microscopy confirmed a cobblestone epithelial morphology with a minor fibroblast-like population (arrows). All cells expressed the epithelial marker EpCAM (green; nuclei: DAPI, blue). (**C**) Western blot confirmation of E6 and E7 oncoprotein expression in TY cells. (**E**) Karyotype analysis at passage 28 revealed two chromosomal patterns: (**left**) 44, XX, -2, -22 and (**F**) (**right**) 44, XX, 5p-, -19, -21, -22, +ring chromosome. Scale bars: 100 µm (**B**), 20 µm (**D**).

After immortalization with E6/E7 and *hTERT*, the established TY cells at passage 90 exhibit a predominantly cobblestone epithelial morphology, with a minor fibroblast-like subpopulation (Figure 1A,B). All cells expressed the epithelial marker EpCAM (Figure 1C). Immunoblot analysis confirmed stable expression of E6 and E7 oncoproteins (Figure 1D). Karyotype analysis at passage 28 revealed two different polyploidic karyotypes. One karyotype was 44, XX, -2,-22 and the other was 44, XX, 5p-, -19, -21, -22, +ring chromosome (Figure 1E).

### 3.2. Optimization of Transformation Detection Capacity

**Figure 2:**
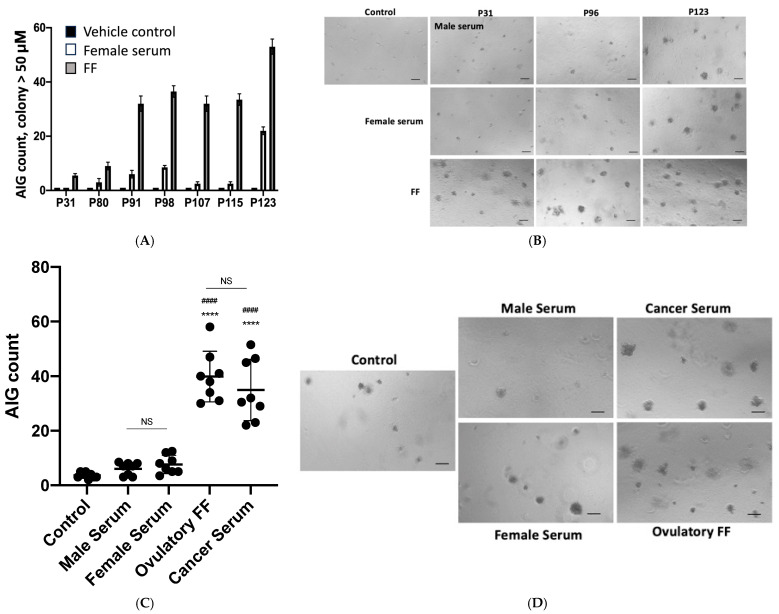
Passage-dependent sensitivity of TY cells in transformation assays. (**A**,**B**) AIG response of TY cells at indicated passages was tested to: pooled ovulatory follicular fluid (FF; *n* = 10, positive control), normal female serum (*n* = 8), or vehicle control. Shown are quantification of mean colonies ± SD from duplicate experiments (**A**), and representative colony images (**B**). (**C**,**D**) TY cells at passage 96 were tested for AIG in responding to treatment of ovulatory FF (*n* = 8), stage 1 breast cancer serum (*n* = 8), and control serum of male and female (*n* = 8 each) and vehicle control. Shown are colony number of each sample and the median (**C**), and representative colony images (**D**). Statistical significance versus male serum (**** *p* < 0.0001) or female serum (^####^ *p* < 0.0001); NS, not significant. Scale bar: 50 µm (all panels).

Through systematic evaluation of multiple passages, we identified passage 96 TY cells as exhibiting optimal responsiveness to transformation signals. This determination was based on their enhanced ability to discriminate between the known transforming FF (from 8 follicles) and control serum (from 8 female and 8 male donors, Table 1). FF, which we previously demonstrated possesses strong transformation potential (16), induced robust AIG in TY cells. Comparing TY cells at different passages, the difference between FF and normal serum was significantly higher for passage 91 to passage 115 than that observed in earlier and later passages (Figure 2A,B). Importantly, these cells showed comparable responsiveness to sera from 8 stage I breast cancer patients (Table S1) (39.89 ± 9.2 colonies) versus FFs (34.93 ± 11.22 colonies), while maintaining minimal background activity with normal donor sera (male: 5.81 ± 4.33; female: 4.0 ± 2.5 colonies) (Figure 2C,D). The results indicated TY cells at passage 96 exhibit the highest ability to detect malignant signals from cancer sera.

### 3.3. Comparative Evaluation of Transformation Detection Across Cell Lines

**Figure 3:**
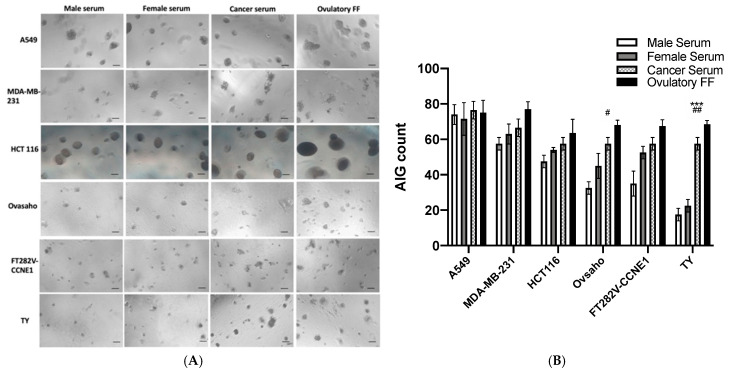
Comparative analysis of AIG responses across cell lines. (**A**) Representative soft agar colonies from four cancer cell lines (A549 [lung], MDA-MB-231 [triple-negative breast], HCT116 [colon], OVSAHO [HGSC]) and two immortalized fallopian tube epithelial (FTE) lines (FT282-CCNE1, TY) exposed to pooled normal serum, cancer serum, or FF. Scale bar: 50 µm. (**B**) Quantified AIG colonies (mean ± SD of triplicate experiments). TY cells showed the greatest differential response between cancer serum (57.50 ± 3.55 colonies) and normal sera (male: 17.50 ± 3.54; female: 22.50 ± 3.54). ^#^ *p* < 0.05, ^##^ *p* < 0.01, comparing cancer vs. male group; *** *p* < 0.001, comparing cancer vs. female group.

We conducted comprehensive comparisons of transformation detection capacity across various established cancer cell lines (A549 lung cancer, MDA-MB-231 triple-negative breast cancer, HCT116 colon cancer) and ovarian cancer-relevant lines (OVSAHO high-grade serous ovarian cancer, FT282-CCNE1, and TY fallopian tube epithelial cells) (Figure 3). FT282-CCNE1 cells—which carry both *TP53* and *CCNE1* alterations—as well as all tested cancer cell lines, displayed constitutive AIG activity irrespective of serum source. Notably, TY cells showed the most pronounced differential response between normal (*n* = 16) and stage 1 breast cancer (*n* = 8) sera (Figure 3). Quantitative analysis revealed that TY cells formed 17.50 ± 3.54 colonies with normal male serum, 22.50 ± 3.54 with normal female serum, and 57.50 ± 3.55 with breast cancer serum—approaching the positive control response to FF (68.50 ± 2.12 colonies). This superior discriminative capacity highlighted TY cells’ unique suitability for serum-based cancer detection.

### 3.4. Diagnostic Performance in Clinical Ovarian Cancer Detection

**Figure 4:**
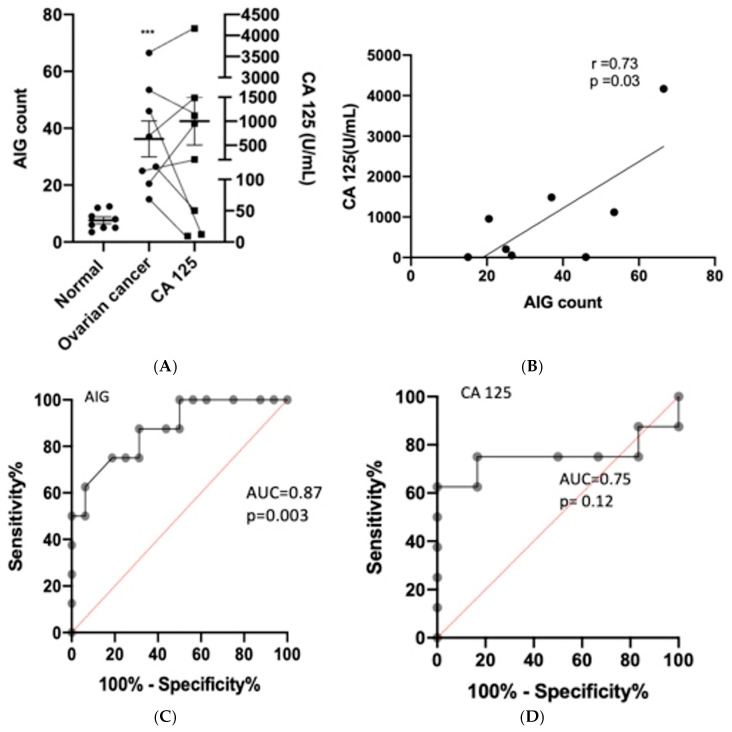
Diagnostic performance of the TY-AIG assay in ovarian cancer detection. (**A**) Colony numbers in TY-AIG assay of ovarian cancer serum (*n* = 8) and normal female control (*n* = 8). (**B**) Scatter plot comparing TY-AIG colony counts and CA125 levels, showing positive correlation between AIG colonies and CA125 levels (Pearson *r* = 0.73, *p* = 0.03). (**C**,**D**) ROC curve analysis demonstrating superior diagnostic accuracy for TY-AIG (AUC = 0.87, *p* = 0.003) versus CA125 (AUC = 0.75, *p* = 0.12). At cutoff = 23.75 colonies, TY-AIG achieved 75% sensitivity and 81% specificity. *** *p* < 0.001.

In clinical validation studies using serum samples from ovarian cancer patients and non-cancer controls (demographics in Table 1 and Table 2), TY cells exhibited significantly higher AIG colony formation with cancer sera (37.06 ± 18.03, range: 15.00–66.50 colonies) compared to control sera (4 ± 2.5 colonies, range: 1.00–8.50 colonies) (Figure 4A). This transformation activity showed strong positive correlation with serum CA125 levels (*r* = 0.73, *p* = 0.03; Figure 4B), suggesting that the AIG assay captures clinically relevant malignant features. Receiver operating characteristic (ROC) analysis demonstrated superior diagnostic performance of the TY-AIG assay (AUC = 0.87, *p* = 0.003) compared to CA125 alone (AUC = 0.75, *p* = 0.12) (Figure 4C,D), indicating its potential as a more accurate diagnostic tool.

### 3.5. Correlation with Disease Burden in Preclinical Models

**Figure 5:**
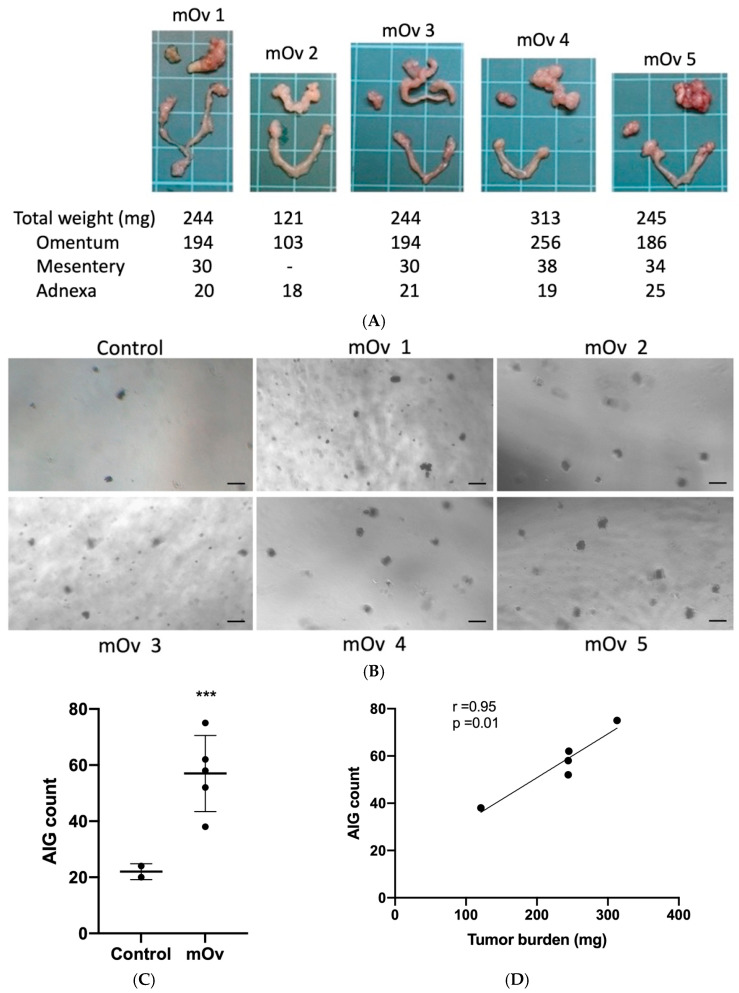
TY-AIG activity reflects tumor burden in ID8 syngeneic ovarian cancer mice. (**A**) Intraperitoneal tumors and weights from five ID8 tumor-bearing mice (mOv-1 to mOv-5), showing predominant omental involvement. (**B**) Representative AIG colonies induced by mouse sera. (**C**) Quantification revealed significantly higher AIG activity in tumor-bearing versus control mice (*** *p* < 0.001, unpaired *t*-test). (**D**) Strong correlation between AIG counts and total tumor volume (*r* = 0.95, *p* = 0.01). Scale bars: 50 µm (**B**), 1 cm (**A**).

To evaluate the assay’s ability to reflect disease progression, we employed an ID8 syngeneic mouse model of ovarian cancer peritoneal dissemination [18]. Four months post-injection, all mice developed characteristic peritoneal metastases, predominantly in the omentum with smaller deposits in the mesentery and adnexa (Figure 5A). Serum from tumor-bearing mice induced significantly higher TY cell AIG (57.0 ± 13.56 colonies) compared to normal mouse serum (22.0 ± 2.83 colonies; Figure 5B,C). Most importantly, AIG counts showed remarkable correlation with total tumor weight (*r* = 0.95, *p* < 0.01) and metastatic burden at specific sites (Figure 5D and Figure S1), suggesting the assay’s potential for disease monitoring.

### 3.6. Mechanistic Insights into Transformation Signaling

**Figure 6:**
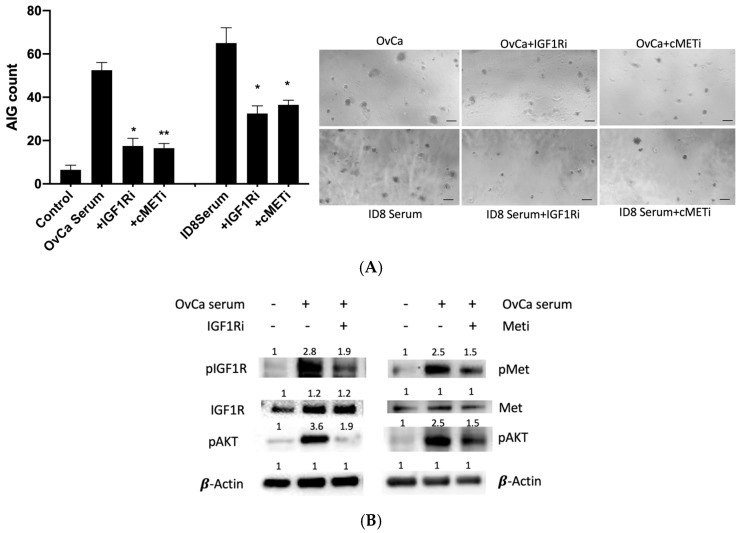
IGF and HGF signaling mediate cancer serum-induced AIG in TY cells. (**A**) TY-AIG colony formation in response to human ovarian cancer (OvCa) serum or mouse ID8 tumor serum, with or without IGF-1R inhibitor (PPP, 100 nM) or c-MET inhibitor (AMG337, 10 µM). Data shown as mean ± SD (*n* = 3); * *p* < 0.05, ** *p* < 0.01 vs. untreated control by two-way ANOVA. (**B**) Western blot analysis of IGF-1R, c-MET, and AKT phosphorylation in TY cells treated with pooled ovarian cancer serum ± inhibitors IGF1Ri: 100 nM [PPP]; cMETi: 10 µM [AMG377]. β-actin served as loading control.

Investigation of the molecular mechanisms underlying serum-induced transformation revealed critical roles for HGF/c-MET and IGF/IGF-1R pathways. Using pooled human and murine ovarian cancer sera, we observed that pharmacological inhibition of c-MET (AMG337) and IGF-1R (PPP) significantly attenuated AIG colony formation (Figure 6A). Western blot analysis demonstrated that cancer serum induced 2.8-fold and 2.5-fold activation of IGF-1R and c-MET autophosphorylation, respectively, with corresponding downstream AKT phosphorylation. These activation patterns were reduced by 30–50% with pathway-specific inhibitors (Figure 6B), confirming these receptors as key mediators of the observed transformation activity.

## 4. Discussion

The present study establishes the TY-AIG assay as a sensitive and specific biotest for detecting cancer-associated transforming activity in serum, offering a novel approach to ovarian cancer diagnosis by measuring anchorage-independent growth (AIG), a hallmark of malignancy.

### 4.1. Development and Optimization of TY Cells

TY cells, generated through sequential transduction of fallopian tube epithelial cells with HPV16 E6/E7 and hTERT, exhibited progressive genomic instability [13], culminating in heightened sensitivity to cancer serum-derived transforming signals. Notably, passage 96 TY cells demonstrated optimal discriminative capacity, forming significantly more AIG colonies in response to cancer sera (68.50 ± 2.12) compared to normal female (17.50 ± 3.54) and male (22.50 ± 3.54) sera (Figure 2B). Beyond passage 123, TY cells lost specificity, responding excessively even to normal sera, underscoring the importance of passage number standardization for diagnostic consistency. Table 1 showed AIG count of non-cancer female and male. Comparative testing of other precancerous and cancerous cell lines (e.g., A549, MDA-MB-231, FT282V-CCNE1) revealed that most either failed to discriminate cancer sera or exhibited constitutive AIG activity. In contrast, TY cells uniquely distinguished cancer from normal sera, supporting their utility as a selective biosensor.

### 4.2. Advancing Cancer Diagnostics Beyond Traditional Biomarkers

Serum tests for solid tumors (e.g., ovarian cancer CA125) rely on indirect markers with limited early-stage sensitivity and specificity due to interference from benign conditions. This contrasts with hematologic malignancies like multiple myeloma, where secreted proteins (e.g., M-proteins [4]) serve as direct indicators. The TY-AIG assay bridges this gap by quantifying functional transforming activity—a direct malignancy readout. Clinically, TY-AIG correlated significantly with CA125 (*r* = 0.73, *p* = 0.03) and outperformed it in ROC analysis (AUC 0.87 vs. 0.75), achieving 75% sensitivity and 81% specificity at a cutoff of 23.75 colonies. Critically, the induction of AIG by sera from ovarian and breast cancer patients (Figure 3B) and ovarian cancer mice (Figure 5C) indicates that systemic release of oncogenic factors is a conserved feature of cancer across species.

### 4.3. Mechanistic Basis of TY-AIG: HGF/c-MET and IGF/IGF-1R Signaling

Early mechanistic studies identified key receptor tyrosine kinase (RTK) pathways—specifically IGF2/IGF-1R, HGF/cMET, and EGFR signaling—as mediators of follicular fluid (FF)-induced transformation in fallopian tube epithelial cells [13,14,15]. Building on this, the present study demonstrates the critical role of cMET and IGF-1R signaling in cancer serum-induced anchorage-independent growth (AIG). This finding aligns with established clinical evidence in ovarian cancer: elevated serum HGF levels decrease upon chemotherapy [19], and tumor tissues exhibit a three-fold increase in IGF2 expression compared to normal tissue, correlating with poorer prognosis [20]. The broader oncological relevance of IGF and HGF signaling is well-documented [21,22]. For instance, circulating IGF2 levels associate with increased risk of ER-positive breast cancer [23], while tissue HGF overexpression correlates with lymph node metastasis and serves as a prognostic indicator in breast cancer [24]. Collectively, these observations suggest that the systemic release of AIG-promoting growth factors is a common feature of malignancy. The TY-AIG assay quantitatively measures this transforming activity, positioning it as a promising diagnostic tool with potential prognostic applications across cancer types.

### 4.4. Limitations and Future Directions

Although the TY cell AIG assay demonstrates considerable diagnostic potential, several limitations should be noted. First, the modest sample size of both cancer and control cohorts, combined with the hospital-based case–control design, may limit the generalizability of our findings to broader populations. Second, potential confounding factors such as menstrual cycle phase, endometriosis associated tissue injury, or other physiological stressors were not accounted for in the current study, and their potential influence on AIG measurements remains to be determined. These limitations highlight the need for: (1) larger-scale, population-based validation studies; (2) systematic investigation of physiological variables affecting assay performance; and (3) standardization of testing protocols across multiple research centers to establish reproducibility.

## 5. Conclusions

In conclusion, we have developed a novel TY cell-based bioassay that sensitively detects malignant transformation activity in human serum through quantification of AIG. The strong correlation between AIG activity and both established biomarkers (e.g., CA125) and actual tumor burden substantiates the clinical utility of this approach. Mechanistically, the assay captures fundamental oncogenic processes mediated by IGF and HGF signaling pathways. These features, combined with the assay’s robust performance characteristics, suggest it may have broad applicability for cancer detection beyond ovarian cancer, pending further validation. Future studies should focus on expanding its clinical implementation while addressing the current technical and methodological limitations.

## Figures and Tables

**Table 1:** Demographics of non-cancer blood donors and the TY AIG results.

Subject ID	Age	Sex	Diagnosis	AIG Count *
210001	42	F	Leiomyoma of uterine cervix	12.0
210002	30	F	Female Infertility	4.0
210003	48	F	Fibroma of ovary	7.0
210004	36	F	Female Infertility	8.5
150128	40	F	Adenomyosis of uterus	5.5
210006	34	F	Female Infertility	4.5
210007	50	F	Female Infertility	1.0
210008	23	F	Adenomyosis of uterus	1.0
210011	32	M	Healthy donor	7.0
210012	34	M	Healthy donor	4.0
210013	44	M	Healthy donor	3.0
210014	37	M	Healthy donor	8.5
210015	39	M	Healthy donor	3.0
210016	44	M	Healthy donor	4.0
210017	48	M	Healthy donor	1.0
210018	34	M	Healthy donor	1.5

* Mean value of duplicate experiment

**Table 2:** Clinical profile and TY-AIG result of ovarian cancer patients.

Subject ID	Age	Staging	Histology	CA125 (U/mL)	AIG Count *
140112	56	IIIB (T3bN0M0)	High-grade serous carcinoma	954.2	20.5
140150	54	IIIC (T3cN1M0)	High-grade serous carcinoma	1486.2	43.5
140155	46	IA (T1aN0M0)	Clear cell carcinoma	50.06	26.5
150031	40	IA (T1aN0M0)	Granulosa cell carcinoma	9.47	46.0
150047	57	IA (T1aN0M0)	Clear cell carcinoma	12.32	15.0
150122	76	IIIC (T3cN0M0)	High-grade serous carcinoma	204.97	25.0
150167	70	IIIC (T3cN1M0)	High-grade serous carcinoma	4167	66.5
150187	57	IIIC (T3cN0M0)	High-grade serous carcinoma	1117.7	53.5

* Mean value of duplicate experiments

## Data Availability

The original contributions presented in this study are included in the article/Supplementary Material. Further inquiries can be directed to the corresponding author.

## Supplementary Materials

The following supporting information can be downloaded at: https://www.mdpi.com/article/10.3390/diagnostics15151975/s1, Table S1. Clinical profile and serum TY-AIG results of breast cancer patients. Figure S1. Site-specific correlation between tumor burden and serum AIG activity in ID8 intraperitoneal model.

## Abbreviations

The following abbreviations are used in this manuscript:

FF	Ovulatory follicular fluid
AIG	anchorage-independent growth
HGF	Hepatocyte growth factor
IGF	Insulin-like growth factor
EGF	Epidermal growth factor
cMET	mesenchymal–epithelial transition factor
EpCAM	expressed the epithelial marker
HGSC	High-grade serous carcinoma
