# Supplementary material for: A Novel Serum-Based Bioassay for Quantification of Cancer-Associated Transformation Activity: A Case–Control and Animal Study
Source: Diagnostics (Basel). 2025 Aug 6;15(15):1975. doi: 10.3390/diagnostics15151975 (PMC12345745; doi:10.3390/diagnostics15151975)
Supplement: Supplementary file 1 [file diagnostics-15-01975-s001.zip › Table S1.pdf]

Table S1. Clinical profile and serum TY-AIG results of breast cancer patients

Patient ID	Age	Site	Type	TNM stage	ER	PR	HER2	AIG*
TCB-00175	44	Lt	Invasive ductal carcinoma	T1L0M0	Positive	Negative	Negative	51.5
TCB-00179	45	Rt	Invasive ductal carcinoma	T1L0M0	Negative	Negative	Negative	29
TCB-00181	56	Lt	Invasive ductal carcinoma	T1L0M0	Positive	Negative	Negative	30.5
TCB-00198	43	Lt	Invasive ductal carcinoma	T1L0M0	Positive	Positive	Positive	45
TCB-00208	59	Lt	Invasive ductal carcinoma	T1L0M0	Positive	Positive	Positive	46.5
TCB-00209	58	Lt	Invasive ductal carcinoma	T1L0M0	Positive	Negative	Negative	23
TCB-00210	36	Lt	Invasive ductal carcinoma	T1L0M0	Negative	Positive	Negative	32
TCB-00190	48	Rt	Invasive ductal carcinoma	T1L0M0	Positive	Negative	Negative	22

*Mean value of duplicate experiment
