# Supplementary material for: A Novel Serum-Based Bioassay for Quantification of Cancer-Associated Transformation Activity: A Case–Control and Animal Study
Source: Diagnostics (Basel). 2025 Aug 6;15(15):1975. doi: 10.3390/diagnostics15151975 (PMC12345745; doi:10.3390/diagnostics15151975)
Supplement: Supplementary file 1 [file diagnostics-15-01975-s001.zip › Figure S1.pdf]

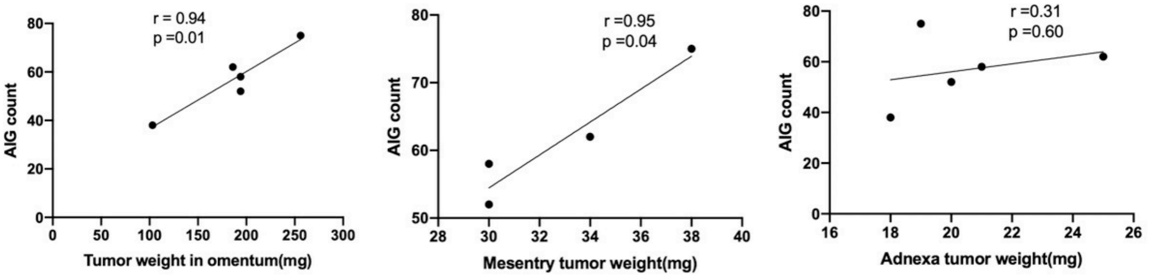

Figure S1. Site-specific correlation between tumor burden and serum AIG activity in ID8 intraperitoneal model.

Quantitative analysis revealed that serum AIG-promoting activity showed stronger correlation with tumor volume in the omentum (Pearson $r = 0.91$, $p = 0.012$) and mesentery ($r = 0.87$, $p = 0.023$) compared to adnexal tumors ($r = 0.62$, $p = 0.19$). Each data point represents an individual tumor-bearing mouse (n=5). Dashed lines indicate 95% confidence intervals.
